# Mushroom polysaccharides with potential in anti-diabetes: Biological mechanisms, extraction, and future perspectives: A review

**DOI:** 10.3389/fnut.2022.1087826

**Published:** 2022-12-14

**Authors:** Xiaofei Liu, Donghui Luo, Jingjing Guan, Jin Chen, Xiaofei Xu

**Affiliations:** College of Food Science and Engineering, Guangdong Ocean University, Yangjiang, China

**Keywords:** oxidative stress, anti-oxidation, anti-lipidemia, anti-inflammation, gut microbiota, insulin resistance, structure-bioactivity relationship

## Abstract

Diabetes mellitus (DM) is a global health threat. Searching for anti-diabetic components from natural resources is of intense interest to scientists. Mushroom polysaccharides have received growing attention in anti-diabetes fields due to their advantages in broad resources, structure diversity, and multiple bioactivities, which are considered an unlimited source of healthy active components potentially applied in functional foods and nutraceuticals. In this review, the current knowledge about the roles of oxidative stress in the pathogenesis of DM, the extraction method of mushroom polysaccharides, and their potential biological mechanisms associated with anti-diabetes, including antioxidant, hypolipidemic, anti-inflammatory, and gut microbiota modulatory actions, were summarized based on a variety of *in vitro* and *in vivo* studies, with aiming at better understanding the roles of mushroom polysaccharides in the prevention and management of DM and its complications. Finally, future perspectives including bridging the gap between the intervention of mushroom polysaccharides and the modulation of insulin signaling pathway, revealing structure-bioactivity of mushroom polysaccharides, developing synergistic foods, conducting well-controlled clinical trials that may be very helpful in discovering valuable mushroom polysaccharides and better applications of mushroom polysaccharides in diabetic control were proposed.

## 1 Introduction

Mushrooms have been a part of human diet for thousands of years for their characteristic flavor and texture as an authentic delicacy. There are approximately 150,000–160,000 mushroom species on earth, of which only 10% are known to science (1). About 700 mushroom species have pharmacological activities and are edible (1). Since the 1970s, global mushroom production has increased more than 30 times, and China has become the world’s largest producer (2). Mushrooms not only be consumed as foods, but also be widely used as medicinal materials. Accumulative evidences from animal models and human intervention studies have demonstrated their bioactivities in anti-cancer (3), anti-inflammation (4), immunomodulation (5), and their ability in the prevention and management of metabolic-related diseases, including diabetes, obesity, and cardiovascular diseases (6), and their protective action on the degenerative brain function (7). Furthermore, some researchers have also proposed the potential of mushrooms in the COVID-19 treatment of the disease COVID-19 (8).

Mushrooms are rich in protein and carbohydrates, with approximately 13–62% of crude protein and 14–75% of carbohydrates in the dry matter for wild-growing mushrooms, whereas they have low content in fat, approximately 0.1–8% (9). The primary elements in mushrooms include potassium, magnesium, calcium, and sodium, with potassium as the dominant element. Vitamins including ascorbic acid, B-group vitamins, tocopherols, and provitamin of ergocalciferol (vitamin D2), pigments, and phenolics, are also widely reported to be present in mushrooms (9). Studies have revealed many types of active components present in mushrooms, including high molecular weight components such as polysaccharides and polysaccharide-protein complexes, lectins, fungal immunomodulatory proteins (FIPs), ribosome-inactivating proteins (RIPs), ribonucleases, laccases, polyphenols, and low molecular weight components such as triterpenes, ergothioneine, alkaloids (5, 6). Among these components, polysaccharides and polysaccharide–protein complexes are highly contained in mushrooms, which primarily contribute to the bioactivities of mushrooms (10).

Diabetes mellitus (DM) is a non-communicable disease characterizing chronic hyperglycemia caused by impaired functions in insulin secretion and/or insulin action (11). Clinically, DM is classified into type 1 diabetes mellitus (T1DM), type 2 diabetes mellitus (T2DM), gestational DM, and specific types of DM due to gene, drug, or disease (12). T2DM is due to a progressive insulin insufficiency on the background of peripheral insulin resistance, accounting for more than 90% of all diabetes (12). DM is a major reason for the development of cardiovascular diseases, blindness, kidney failure, and amputations of limbs worldwide, which are called complications (13). In 2017, 6.28% of the world’s population was affected by T2DM, and the global prevalence rate of T2DM is estimated to be approximately 7.08% by 2030 (14). The high prevalence of DM and its complications place a tremendous burden on the healthcare system worldwide. The pharmacological mechanisms of drugs for the management of DM include inhibition of glucose absorption from diets, reduction of hepatic glucose production, and increased insulin sensitivity in tissues (15). However, currently available most of the synthetic drugs in the long-term management of DM have side effects such as weight gain and hypoglycemia along with their therapeutic potential (15). Natural components derived from food materials and traditional herbal medicines, such as mushrooms, and medicinal plants, have emerged as safe and relatively economical approaches (16, 17).

Interdisciplinary studies focusing on mushrooms have documented much of the knowledge on mushroom polysaccharides and increasingly demonstrate valuably pharmaceutical properties of mushroom polysaccharides extracted from a number of species. As a result, several reviews have been conducted in the field of mushroom polysaccharides related to DM. Khursheed et al. (18) have summarized mushroom polysaccharides with anti-diabetic activity mainly derived from *Hericium erinaceus*, *Phellinus linteus*, *Inonotus obliquus*, *Catathelasma ventricosum*, and *Ganoderma lucidum* from the view of modulating gut microbiota. The potential applications of mushroom polysaccharides in diabetic complications have also been proposed by researchers (19). Additionally, mushroom polysaccharides ameliorating oxidative stress, beta-cell dysfunction, and insulin resistance, which are closely associated with DM, through antioxidant action have been discussed recently (20). In this review, the extraction methods of mushroom polysaccharides, and mushroom polysaccharides with potentials in the management of DM reported in recent 3 years were summarized; the biological mechanisms including anti-oxidation, hypolipidemia, anti-inflammation, and modulating gut microbiota in anti-diabetes, and future perspectives were also discussed for a better understanding the current status of knowledge in such fields and providing a valuable reference for the development and application of mushrooms polysaccharides in functional foods and nutraceuticals for DM and its complications management.

## 2 Pathogenesis of diabetes mellitus and its complications

**FIGURE 1:**
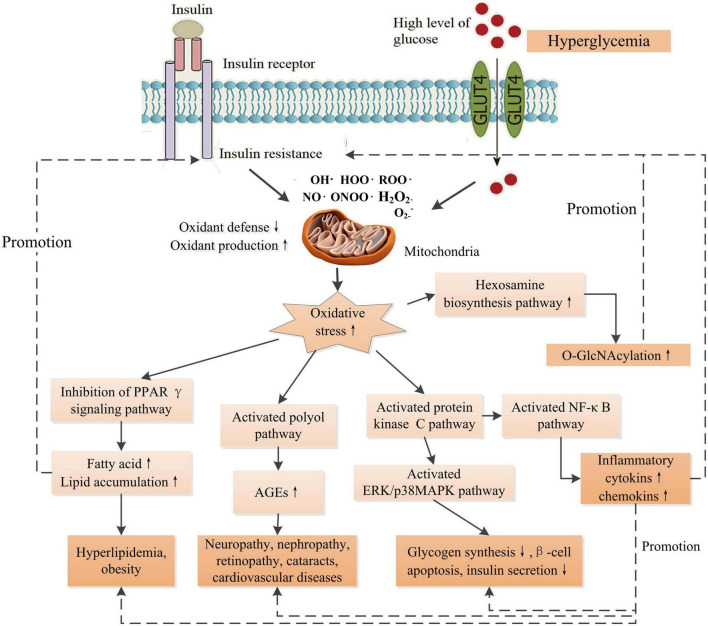
Schematic diagram of the effects of increased oxidative stress associated with insulin resistance and high glucose level on the signaling pathways of the development of DM and its complications. O-GlcNAcylation could promote the production of AGEs. AGEs contribute to inflammation through NF-κB signaling pathway. There are intricate links between oxidative stress and the development of complications. PPARγ, peroxisome proliferator activated receptor-γ; AGEs, advanced glycation end-products; ERK, extracellular-signal-regulated kinase; MAPK, mitogen-activated protein kinase; O-GlcNAcylation, O-linked-acetylglucosamine. ↑ Indicates increase and ↓ indicates decrease.

Reactive oxygen species (ROS) and reactive nitrogen species (RNS) are vital for normal physiology in human body. The ROS/RNS participate in normal physiology, including reversible protein modifications, adaptive mitogen-activated protein kinase (MAPK) signaling activation, and modulation of gene regulation, and play essential roles in a number of physiological processes. Therefore, cells keep a physiological content of ROS/RNS to maintain homeostasis (21). Endogenous factors, such as hormones, and pro-inflammatory cytokines, and exogenous factors, such as nutrients, and ultraviolet irradiation, trigger ROS/RNS generation through multiple mechanisms, including mitochondria, NADPH oxidase, nitric oxide synthase, etc. (22). High metabolic load such as elevated glucose and/or free fatty acid levels, inflammation, ER stress, and endocrine dysregulation, increase the production of ROS/RNS in cells (22). Oxidative stress is a result of the overproduction of ROS/RNS mainly produced by mitochondria, leading to an imbalance between antioxidants and pro-oxidants. This means excessive production and/or incapable removal of ROS/RNS within cells (23). A growing number of *in vitro* and *in vivo* studies have proposed that oxidative stress plays a central role in the pathogenesis of DM and its complications (Figure 1). Oxidative stress disrupts normal insulin signaling and progressively induces insulin resistance (22). Insulin resistance is an early symptom of the pathogenesis of DM characterized by high levels of blood insulin and glucose, defined as prediabetes. High level of insulin damages to β-cell mass and/or function progressively. Moreover, abnormal insulin level modulates the expressions of key molecules such as proprotein convertase subtilisin/kexin type 9 (PCSK9), low-density lipoprotein receptor (LDLR) in lipid metabolism, and causes disorders of lipid metabolism (13). Additionally, oxidative stress-induced damages to DNA, proteins, and lipids in cells impair the structures and functions of cells and organs. Cumulative damages over time ultimately result in the pathogenesis of a variety of diseases including DM, cancer, Alzheimer’s disease, chronic renal failure, etc. (23). Furthermore, high level of glucose compromise autophagy in endothelial cells, leading to endothelial dysfunction and inducing disorder of platelets and thrombosis (13), therefore, causing cardiovascular diseases ultimately such as coronary artery disease and microvascular diseases such as nephropathy, neuropathy, and retinopathy. Therefore, once hyperglycemia occurs, endogenous and exogenous factors drive patients to develop multiple chronic complications eventually.

## 3 Polysaccharides from mushrooms with anti-diabetes potentials

Polysaccharides from medicinal plants, grains, fruits, vegetables, edible mushrooms, and medicinal foods have a wide range of bioactivities such as hypoglycemic, hypolipidemic, antioxidant, anti-inflammatory, and prebiotic effects, which are associated with anti-diabetes (24). Mushroom polysaccharides are naturally active components consumed frequently in our lives. Due to their low toxicity, easy availability, and multiple bioactivities, the evaluation of mushroom polysaccharides in anti-diabetic potential has attracted extensive interest from researchers (18, 24). The frequently investigated mushrooms include *Cordyceps militaris*, *H. erinaceus*, *P. linteus*, *I. obliquus*, *C. ventricosum*, *G. lucidum*, and *Grifola frondosa*. In recent years, a number of novel polysaccharides derived from a variety of mushrooms such as *Auricularia auricula*, *Auricularia polytricha*, and *Dictyophora indusiata* have been documented to have multiple pharmacological properties associated with anti-diabetic potentials, including improving lipid metabolism, increasing hepatic glycogen synthesis, anti-inflammation, and reducing insulin resistance, etc. (Table 1).

**TABLE 1:** Polysaccharides derived from mushrooms with anti-diabetic potentials reported in recent years.

Mushroom resource	Structure characteristics	Model	Doses of administration	Outcomes	References
*Auricularia auricula*	AAP polysaccharides consisted of fucose, glucose, galactose, xylose, rhamnose, and mannose with a molecular weight of 173 kDa.	STZ-induced diabetic mice	Oral administration of 100 and 300 mg/kg, respectively, for 4 weeks.	Blood glucose ↓, serum insulin ↑, CAT ↑, MDA ↓.	(25)
*Auricularia polytricha*	APP polysaccharides consisted of fucose, glucose, galactose, xylose, rhamnose and mannose with a molecular weight of 17.1 kDa.	STZ-induced diabetic mice	Oral administration of 100 and 300 mg/kg, respectively, for 4 weeks.	Blood glucose ↓, serum insulin ↑, TNF-α↓, SOD ↑, CAT ↑, MDA ↓.	(25)
*Amillariella mellea*	Alkaline soluble and neutral mannogalactoglucan.	db/db mice	Oral administration of 50 mg/kg/d BW for 4 weeks.	Blood glucose ↓, insulin sensitivity ↑, reversed impaired glucose tolerance. Improved hepatic lipid metabolism and injury. Protecting pancreatic islets from compensatory enlargement.	(26)
*Agrocybe cylindracea*	Heteropolysaccharides composed of mannose, ribose, rhamnose, glucuronic acid, galacturonic acid, glucose, galactose, xylose, arabinose, and fucose.	HFD and STZ-induced T2DM mice	Oral administration of 100, 200, and 400 mg/kg/d BW, respectively, for 4 weeks.	Blood glucose ↓, liver and colon injuries ↓, inflammation ↓, SOD ↑, GSH-Px ↑, CAT ↑. Restoration of lipid metabolism.	(27)
*Cordyceps militaris*	Polysaccharides with a backbone of (1→4)-β-D-Glcp and (1→2)-α-D-Manp and with a molecular weight of 700 kDa.	LDLR^(–/–)^ mouse fed with a high-fat and-cholesterol diet	Oral administration of 50, and 100 mg/kg/d BW, respectively, for 8 weeks.	Reduced atherosclerotic plaque formation and serum TC ↓, TG ↓, HDL-C ↑. Improved apolipoprotein levels.	(28)
*Cordyceps militaris*	Polysaccharides-protein complex without precise characterization.	HFD-induced obese mice	Oral administration of 100 mg/kg/d BW for 8 weeks.	Adipocyte size and liver steatosis ↓. No effect on SCFAs production. Diversity ↑ and richness ↑ in gut microbiota. Relative abundance of Lactobacillus ↓, Dorea ↓, Clostridium ↓, Ruminococcus ↑, Akkermansia ↑.	(29)
*Cordyceps militaris*	Selenium-rich polysaccharides-protein complex without precise characterization.	HFD-induced obese mice	Oral administration of 50, 100, and 200 mg/kg/d, respectively, for 8 weeks.	Adipocyte size and liver steatosis ↓. Serum TG ↓, LDL-C ↓. No effect on SCFAs production. Diversity ↑ and richness ↑ in gut microbiota. Relative abundance of Lactobacillus ↓, Dorea ↓, Clostridium ↓, Ruminococcus ↑, Akkermansia ↑.	(29)
*Dictyophora indusiata*	Crude polysaccharides composed of glucose, mannose, and galactose.	HFD-induced obese mice	Oral administration of 200 and 400 mg/kg/d, respectively, for 4 weeks.	Fat accumulation ↓. Serum TG ↓, free fatty acid ↓, glucose ↓, insulin ↓. Intestinal barrier function ↑ and liver injury ↓. TNF-α↓, IL-1β↓, and IL-6 ↓ and IL-4 ↑ and IL-10 ↑. Diversity ↑ and richness ↑ in the gut microbiota. Decreased ratio of Firmicutes to Bacteroidetes. Relative abundance of Bacilli ↓, Gammaproteobacteria ↓, and Bacteroidia ↑.	(30)
*Hericium erinaceus*	Polysaccharide (81.51%)-protein (1.97%) complex composed of rhamnose, arabinose, mannose, glucose, and galactose with weight-average molecular weights of 263.6 kDa.	STZ-induced diabetic rats	Oral administration of 150 and 300 mg/kg, respectively, for 4 weeks.	Blood glucose ↓, glucose tolerance ↑, hepatic function and serum lipid metabolism ↑, antioxidant enzyme activity ↑, lipid peroxidation ↓, and glycogen synthesis ↑.	(31)
*Inonotus obliquus*	Polysaccharides consisted of mannose, rhamnose, glucuronic acid, xylose, arabinose, fucose, galacturonic acid, glucose, and galactose with Mw of 373 kDa.	HFD and STZ-induced T2DM mice	Oral administration of 150, 300, and 6 00 mg/kg/d BW, respectively, for 5 weeks	Blood glucose ↓, serum TC ↓, TG ↓, LDL-C ↓, HDL-C ↑. TNF-α↓ and IL-6 ↓. Intestinal barrier function ↑. Increased ratio of Firmicutes to Bacteroidetes. Relative abundance of Akkermansia ↑, Lactobacillus ↑, Parabacteroides ↓, Bacteroides ↓.	(32)
*Ganoderma atrum*	PSG composed of mannose, glucose, galactose, rhamnose, and arabinose with molecular weight of 1013 kDa with	HFD and STZ-induced diabetic rats	Oral administration of 50 mg/kg/d BW for 4 weeks.	Blood glucose ↓, insulin resistance level ↓. TC ↓, TG ↓, and LDL-C ↓ and HDL-C ↑. Protection of islet cells and intestine injuries. Restoration of the decreased ratio of Firmicutes and Bacteroidetes. Relative abundance Prevotella spp ↓, Blautia spp ↓, Streptococcus spp ↓, and Clostridium spp ↓, Lactobacillus spp ↑, Oscillospira spp ↑, Coprococcus spp ↑, and Ruminococcus spp ↑.	(33)
*Ganoderma lucidum*	GLP composed of mannose, glucose, galactose, rhamnose, and arabinose with weight-average molecular weight of 13.7 kDa.	HFD and STZ-induced diabetic rats	Oral administration of 400 mg/kg/d for 4 weeks.	Improved lipid metabolism and inflammation ↓. Blood glucose ↓, insulin ↓. Relative abundance of Aerococcus ↓, Ruminococcus ↓, Corynebacterium ↓, Proteus ↓, Blautia ↑, Dehalobacterium ↓, Parabacteroides ↓, Bacteroides ↓. Restoration of the changes in metabolism of gut microbiota.	(34)
*Ganoderma lucidum*	Spore-derived polysaccharides composed of glucose, mannose, and galactose containing (1→3)-β-D-Glcp, (1→3,6)-β-D-Glcp, (1→6)-β-D-Glcp, and terminal-β-D-Glcp moieties with molecular weight of 26.0 kDa.	HFD-induced obese mice	Oral administration of 100 and 300 mg/kg/d, respectively, for 12 weeks.	Fat accumulation ↓, hepatic steatosis ↓. Serum TG ↓ and HDL-C ↑. No obvious effects on fasting hyperglycemia and glucose intolerance. Reduced ratio of Firmicutes/Bacteroidetes. Relative abundance of Allobaculum ↑, Bifidobacterium ↑, and *Christensenellaceae_R-7_group* ↑. SCFAs levels ↑.	(35)
*Grifola frondosa*	Heteropolysaccharides mainly composed of mannose, rhamnose, glucuronic acid, galacturonic acid, glucose, galactose, and fucose with molecular weight of 18.18 kDa in the main fraction.	HFD and STZ-induced diabetic mice	Oral administration of 300 and 900 mg/kg/d, respectively, for 4 weeks.	Blood glucose ↓, glucose tolerance ↑, TG ↓, LDL-C ↓ in serum and hepatic glycogen level ↑. Liver TC ↓, TG ↓, and free fatty acids ↓. Relative abundance of Alistipes ↑, Streptococcus ↓, Enterococcus ↓, Staphylococcus ↓, and Aerococcus ↓.	(36)
*Grifola frondosa*	Polysaccharides mainly consisted of →4)-α-D-Glcp-(1→, β-D-Glcp-(1→, and →4,6)-β-D-Glcp-(1→ with molecular weight of 5,570 kDa	HFD-fed obese mice	Oral administration of 50, and 100 mg/kg/d, respectively, for 8 weeks.	Serum TG ↓, TC ↓, and LDL-C ↓, HDL-C ↑. Fat accumulation in the liver ↓ and hepatic steatosis ↓. Blood glucose level ↓.	(37)
*Lyophyllum decastes*	Heteropolysaccharides mainly composed of mannose, glucose, galactose, and fucose with linkages of 1,3-Fuc*p*, T-Gal*p*, 1,4-Glu*p*, 1,6-Glu*p*, 1,6-Gal*p*, and 1,2,6-Man*p*.	HFD-fed obese mice	Oral administration of 500, and 1000 mg/kg/d, respectively, for 8 weeks.	Hepatic steatosis ↓, serum TC ↓, HDL-C ↓. TNF-α↓, IL-6 ↓, IL-1β↓. A tendency to reduce the ratio of Firmicutes to Bacteroidetes. Relative abundance of *Bacteroides intestinalis* ↑, *Bacteroides sartorii* ↑, *Lactobacillus johnsonii* ↑.	(38)
*Phellinus linteus*	Crude heteropolysaccharides composed of glucose, arabinose, fucose, galactose, xylose, mannose, and arabinose.	HFD and STZ-induced diabetic rats	Oral administration of 600 mg/kg/d for 8 weeks.	Insulin resistance ↓, fasting insulin levels ↓, and HOMA-IR ↓. CRP ↓, TNF-α↓, and IL-6 ↓. SCFAs content ↑, restored intestinal mucosal layer thickness, and intestinal barrier function ↑. Relative abundance of *Lachnospiraceae-NK4A136* ↑, *Lachnospiraceae-UCG-006* ↑, Roseburia ↑, Prevotella9 ↑, Blautia ↑, *Ruminiclostridium-9* ↑, *Eubacterium xylanophilum* ↑, Anaerotruncus ↑, Oscillibacter ↑, *Clostridium_sensu_stricto_1* ↓, *Escherichia-Shigella* ↓, *Bacteroidales_S24-7_group* ↓, and Akkermansia ↓.	(39)
*Sarcodon aspratus*	Polysaccharides-protein complex composed of mannose, glucose, galactose, and arabinose.	HFD-induced obese mice	Oral administration of 100, 200, and 400 mg/kg/d, respectively, for 14 weeks.	Glucose intolerance ↑, hepatic steatosis ↓, lipid homeostasis ↑. Liver oxidative stress ↓, inflammation ↓, adipocyte differentiation ↓. Decreased ratio of Firmicutes/Bacteroidetes. Relative abundance of Verrucomicrobia ↑, Proteobacteria ↑. Lactobacillus ↑, Bacteroides ↑, Akkermansia ↑.	(40)
*Suillellus luridus*	Polysaccharide with backbone of 1,3-α-D-Galp, 1,3-β-D-Glcp and 1,6-β-D-Glcp composed of galactose, glucose, arabinose, and mannose with molecular weight of 9.4 kDa.	STZ-induced diabetic mice	Oral administration of 100 mg/kg/d for 30 days.	Hepatic glycogen ↑, blood glucose ↓, insulin ↑. Activities of SOD ↑, GSH-Px ↑, CAT ↑ in tissues. Serum TC ↓, TG ↓, LDL-C ↓, and HDL-C ↑.	(41)
*Tremella fuciformis*	Polysaccharides without structure determination.	HFD and STZ-induced diabetic rats	Oral administration of 200 mg/kg/d for 4 weeks.	Blood glucose ↓, serum insulin ↓, TC ↓, HDL-C ↑, LDL-C ↓.	(42)

HFD, high-fat diet; STZ, streptozotocin; TC, total cholesterol; TG, triglyceride; HDL-C, high-density lipoprotein cholesterol; LDL-C, low-density lipoprotein cholesterol; SOD, superoxide dismutase; GSH-Px, glutathione peroxidase; CAT, catalase; FFA, free fatty acid; HMA-IR, homeostatic model assessment for insulin resistance; CRP, C-reactive protein. ↑ Represents increase; ↓ represents decrease.

## 4 Biological mechanisms of mushroom polysaccharides on anti-diabetes effects

### 4.1 Antioxidant action

Oxidative stress arising from the overproduction of ROS/RNS within cells plays a crucial role in the pathogenesis of many chronic diseases, including DM, cancer, cardiovascular diseases, Alzheimer’s disease, and so on. A large number of studies have documented that mushroom polysaccharides have notable activities in scavenging against 1,1-Diphenyl-2-picrylhydrazyl (DPPH), hydroxyl radicals, superoxide radicals, hydrogen peroxide, nitric oxide, and peroxynitrite, and inhibiting lipid peroxidation (43, 44). Among the active components contained in mushrooms, including flavonoids, polysaccharides, phenols, phenolic compounds, tocopherols, ascorbic acid, and terpenes, polysaccharides exhibit relatively low antioxidant activities in terms of scavenging activities against free radicals using *in vitro* models (43). However, the evaluation of scavenging activities *in vitro* is not a fully valid proof to claim the antioxidant effects of the polysaccharides because the scavenging activities investigations cannot simulate the interaction of antioxidants with chemical free radicals in the internal conditions of cells in a complex organism (45). Interestingly, numerous *in vivo* studies have shown that mushroom polysaccharides are capable of protecting the antioxidant activities of enzymes such as superoxide dismutase (SOD), catalase (CAT), and glutathione peroxidase (GSH-Px), which are major components of the defense system against ROS within body (20). For instance, oral administration of *Tremella fuciformis* polysaccharides (TFPS) markedly improved the SOD and GSH-Px activities in serum, liver, and heart tissues in D-galactose-induced aging mice (46). *G. lucidum* polysaccharides showed protective effects against acute liver injury induced by restraint stress in mice, evidenced by the improvement of GSH-Px, CAT, and SOD activities and a decrease in the activities of ALT and AST (47). *Agaricus blazei* Murill polysaccharides alleviated liver and lung damages by improving the activities of SOD, GSH-Px, and CAT to relieve oxidative stress in organ dysfunction syndrome (MODS) mice (48). Moreover, many studies have also revealed that mushroom polysaccharides enhance the activities of antioxidant enzymes including SOD, GSH-Px, and CAT in diabetes models (Table 1). However, the molecular mechanisms by which mushroom polysaccharides modulate the activity of antioxidant enzymes *in vivo* are still unclear. Natural macromolecules such as polyphenols can provide antioxidant protection against oxidative damage by regulating complicated intracellular signals for the induction of antioxidant enzymes (49). Plant-derived polysaccharides have been reported to promote the expression of antioxidant genes in *Drosophila* and cell models (50, 51). Polysaccharides derived from *Suillellus luridus* have been supposed to relieve oxidative stress in STZ-induced diabetic mice by regulating the nuclear factor erythroid 2-related factor 2 (Nrf2)/heme oxygenase-1 (HO-1) pathway (41). Nrf2 modulates the expression of antioxidant and cyto-protective related genes (52). Therefore, more studies would be needed to uncover the molecular mechanism of how mushroom polysaccharides regulate the expression profiles of antioxidant genes in the mammalian models as well as the Nrf2/HO-1 pathway.

### 4.2 Anti-hyperlipidemic action

**FIGURE 2:**
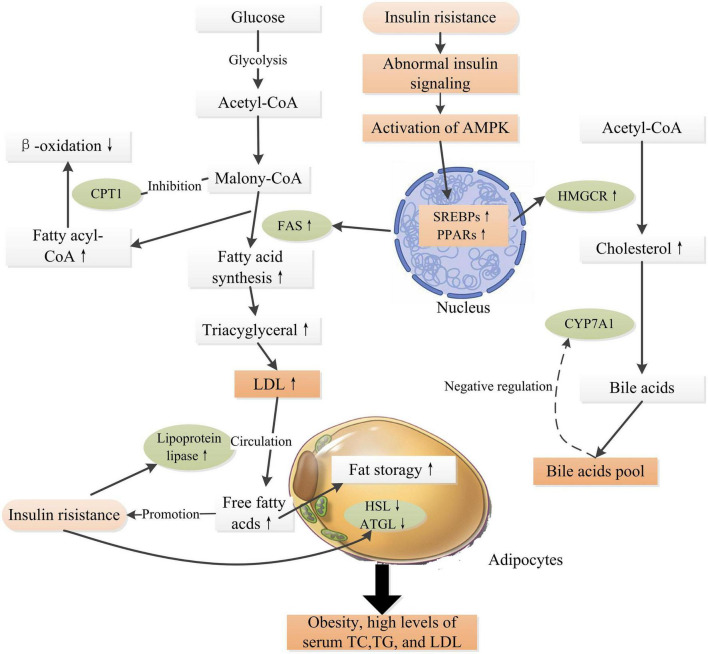
Schematic representation of insulin resistance and high glucose level inducing obesity and hyperlipidemia. Insulin resistance induces abnormal insulin signaling, activating transcription factors such as SREBPs, PPARs, leading to up-expressions of FAS and HMGCR, which promote fatty acid synthesis and cholesterol synthesis. CYP7A1 is a rate limiting enzyme in the pathway of transforming cholesterol into bile acids, which is regulated by the “bile acid pool” within human body. The β-oxidation of long-chain fatty acids is regulated by the activity of CPT1, which is highly sensitive to inhibition by malonyl-CoA (55). In adipose tissue, insulin is an anti-lipolytic hormone that inhibits the actions of HSL and ATGL, thus increasing fatty acid storage in adipocytes. Meanwhile, insulin increases LPL activity, releasing free fatty acids into the circulation from LDL (56). High levels of fatty acids in the circulation further, in turn, deteriorate insulin resistance. Obesity is an independent risk factor for the pathogenesis of DM and related complications. PPARs, peroxisome proliferator activated receptors; AMPK, adenosine (AMP)-protein kinase; SREBPs, sterol regulatory element binding proteins; ACC, acetyl-CoA carboxylase; HMGCR, 3-hydroxy-3-methylglutaryl-CoA reductase; FAS, fatty acid synthase; CYP7A1, cytochrome P450 family 7 subfamily a member 1; CPT1, carnitine palmitoyltransferase-1; HSL, hormone-sensitive lipase; ATGL, adipose triglyceride lipase. ↑ Indicates increase and ↓ indicates decrease.

A high load of free fatty acids can result in reduced glucose uptake and insulin resistance in cells (53), which is a critical factor in the pathogenesis of obesity and type 2 diabetes. Ectopic lipid deposition within cells deteriorates insulin resistance and ultimately accelerates the progression of DM and various chronic complications (54). Intracellular molecules such as peroxisome proliferator activated receptors (PPARs), sterol regulatory element binding protein-1c (SREBP-1c), adenosine (AMP)-protein kinase (AMPK), acetyl-CoA carboxylase (ACC), and 3-hydroxy-3-methylglutaryl-CoA (HMG-CoA) reductase, fatty acid synthase (FAS), cytochrome P450 family 7 subfamily a member 1 (CYP7A1), and carnitine palmitoyltransferase-1 (CPT1) are critical mediators in the regulation of the biosynthesis of fatty acid, triglyceride, and cholesterol, and fatty acid oxidation within cells (55). Experiment evidences have proven that insulin resistance regulates these key mediators and results in abnormal lipid metabolism, progressively leading to obesity and hyperlipidemia, which in turn deteriorate insulin resistance, and therefore accelerate the progression of DM and its complications (Figure 2) (55–57).

Low-density lipoprotein (LDL) receptor-involved endocytosis is vital for cholesterol homeostasis, and upregulation of the LDL receptor results in a decrease in blood LDL-C level. However, in an LDLR^(–/–)^ mouse model, *C. militaris* derived polysaccharides (CM) could decrease plasma total cholesterol (TC) and triglyceride (TG), and increase the high-density lipoprotein cholesterol (HDL-C) and improve apolipoprotein levels. Further molecular mechanical analysis revealed that CM markedly enhanced VLDLR expression and decreased SREBP-1c and ApoB expression at the protein level in the liver, and significantly increased the protein expression of LXRα/ABCG5 in the small intestine, as well as downregulation of the protein expression of PPARγ and ATGL in the epididymal fat (28). This experiment demonstrated a non-LDL receptor-involved cholesterol lowering pathway.

*Pleurotus ostreatus* polysaccharides fraction reduced liver triglyceride level through modulating the expressions of Dgat1, Ldlr, Nr1h4, Acat1, Srebf1, and Srebf2 in diet-induced hypercholesterolemic mice (58). Treatment with *Trametes versicolor* polysaccharide (EVP) dramatically reduced serum TC, TG, LDL-C, and atherosclerosis index in a dose-dependent manner in high-fat diet-induced hyperlipidemic mice. A significant increase in serum lipoprotein lipase (LPL) activity and a notable decrease in protein expression of hepatic 3-hydroxy-3-methylglutaryl-CoA reductase (HMGR) were observed, demonstrated that the anti-lipidemic properties of EVP probably via regulation of hepatic LPL and HMGR (59). *Grifola frondosa* polysaccharides (GFP) were proven to be capable of decreasing the levels of fasting blood glucose (FBG), oral glucose tolerance (OGT), TC, TG, and LDL-C in serum, and reducing the hepatic levels of TC, TG, and free fatty acids through inhibiting the expressions of SREBP-1c, ACC, Cd36, and HMGCR, and promoting the expressions of Acox1, Ldlr, CYP7A1, and BSEP in gene levels in high fat diet (HFD) and STZ-induced diabetic mice (36). *Dictyophora indusiate* crude polysaccharides displayed hypolipidemic actions, including a decrease in fat accumulation and serum TG and free fatty acid levels by inhibiting the expressions of lipogenic genes, such as PPAR-γ, C/EBPα, SREBP-1c, acetyl-CoA carboxylase-1 (ACC-1), and FAS (30). The polysaccharide-protein complex obtained from *Sarcodon aspratus* could improve lipid metabolism in HFD-induced obese mice by regulating lipogenesis meditators (ACC-1 and PGC-1α) (40). The current results suggested that different mushroom polysaccharides might play a role in hypolipidemic action through differential molecular mechanisms. Taken together, these observations demonstrated that mushroom polysaccharides could exert anti-lipidemic action by multiple pathways, including reducing TG and TC synthesis, decreasing lipid storage in adipocytes, elevating energy production, and promoting TC secretions from the liver and small intestine. Further studies are needed to illustrate the detailed mechanism of hypolipidemia of mushroom polysaccharides with high purity and precise structure characterization.

Numerous animal and human clinical trial studies have demonstrated that dietary polysaccharides, such as plant polysaccharides, mushroom polysaccharides, and alga polysaccharides, have potential anti-hyperlipidemic effects (60). It is commonly recognized that oat or barley β-glucan greater than 3.0 g/d is effective in lipid-lowering function in humans, and the high molecular weight of β-glucan is more effective than those with low molecular weight (60). Contrary to intensive investigations of oat or barley β-glucan in clinical trials, the investigation of anti-lipidemic effects of mushroom polysaccharides is mainly carried out in animal models. Thus, clinical trials to assess the efficacy of mushroom polysaccharides in metabolic disorders management are necessary for their successful applications (61). Additionally, structure-bioactivity relationship of mushroom polysaccharides is also an exciting topic, which provides some guides for exploring highly active polysaccharide. For example, degraded polysaccharides obtained from *G. lucidum* with a molecular weight of 13.6 kDa exhibited higher anti-lipidemic effects than the original ones with a higher molecular weight of 3.06 × 10^3^ kDa, including atherosclerosis index, TC, TG, low-density lipoprotein cholesterol (LDL-C), and HDL-C in HFD-induced hyperlipidemia mice (62).

### 4.3 Anti-inflammatory action

Inflammation promotes the pathogenesis of insulin resistance and DM (63). Multiple studies have solidified a link between low-grade chronic inflammation and the development of DM, particularly T2DM, and the deterioration of cardiovascular diseases in people with DM. Obesity and insulin resistance are characterized by a chronic low-grade inflammation (64). Therefore, many factors, such as dysbiosis, intestinal barrier dysfunction, and low-fiber diets, which could aggravate inflammation, may be related to the progression of DM and its complications (65).

In the past several decades, mushroom polysaccharides have attracted growing interest from global researchers due to their outstanding immune-modulatory and anticancer properties and diversity in resources and structures (66). Mushroom polysaccharides are well known as biological response modifier (BRM) and immune-modulatory activity is one of their most important biological properties (67). The immunomodulatory mechanisms of mushroom polysaccharides to immune cells have been well recognized. Mushroom polysaccharides can interact with immune cells such as dendritic cells (DCs), macrophages, and NK cells through cell surface receptors and subsequently activate intracellular cascade signaling to induce immune responses (68). A variety of immune cell receptors, including dectin-1, scavenger receptors, complement receptor 3, lactosylceramide, and toll-like receptors (TLRs), are proposed to be involved in mushroom polysaccharide-induced immune responses, including immune modulation, anticancer, and anti-inflammation (68, 69). For example, TLR-2, TLR-4, and Dectin-1 have been identified to be involved in the activation of immune-related intracellular signaling of a novel polysaccharide from *C. militaris* (70). Natural polysaccharides have been documented to suppress the production of cyclooxygenase 2 (COX2) and inducible nitric oxide synthase (iNOS), which are associated with a reduction in pro-inflammatory responses, and modulate NF-κB-related signaling pathways to increase expression of anti-inflammatory cytokines like IL-10 and decrease expression of inflammatory cytokines such as TNF-α, IL-1β, leading to anti-inflammatory action (71). For instance, TFPS attenuated LPS-induced inflammation through inhibiting miR-155, a small RNA which regulates NF-κB, to inactivate NF-κB signaling pathway, leading to reduce the production of TNF-α and IL-6 in macrophages (72). Observations derived from dextran sulfate sodium (DSS)-treated animal model suggested that the anti-inflammatory effects of mushroom polysaccharides might be gut microbiota-dependent *in vivo* since β-glucan aggravated colitis features of DSS-treated mice which were given drinking water with antibiotics to diminish gut microbiota (73).

**FIGURE 3:**
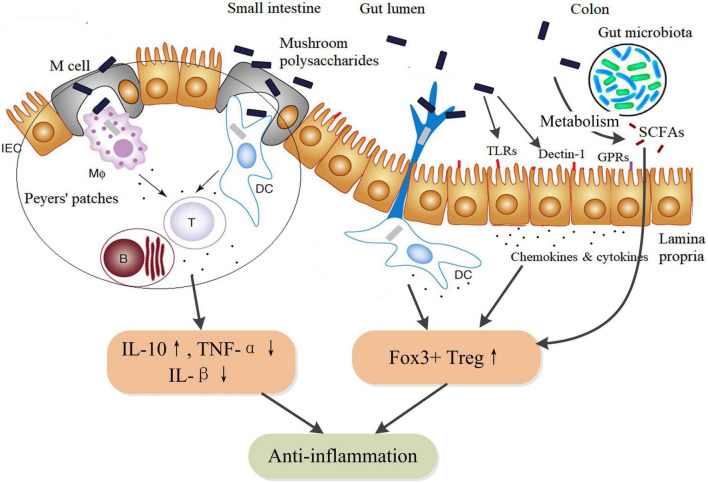
Schematic diagram of the anti-inflammatory action pathways of mushroom polysaccharides and their metabolites SCFAs. SCFAs, such as acetate, butyrate, and propionate, are metabolites of mushroom polysaccharides by fermentation of the gut microbiota. SCFAs facilitate the proliferation and differentiation of IL-10-producing Foxp3+ Treg by inhibiting histone deacetylase (HDAC) activity through G protein–coupled receptors (GPRs). Additionally, butyrate could directly regulate the function of macrophages and dendritic cells to induce Foxp3+ Treg (78). IEC, intestinal epithelial cell; MΦ, macrophages; DC, dendritic cell; B, B lymphocytes; T, T_*H*_ lymphocytes; TLR, toll-like receptors; GPRs, G protein–coupled receptors (GPR41, GPR43, and GPR109A); SCFAs, short chain fatty acids.

Intestinal epithelial cells (IECs) express TLRs and Dectin-1 receptors by which mushroom polysaccharides could activate a variety of physiological responses, including immune regulation, anti-inflammation, and enhanced barrier function (74–76), which might be important action pathways for exerting pharmacological properties of mushroom polysaccharides. In addition, as an essential part of gut-associated lymphoid tissue, Peyer’s patches distribute in the ileum region of the small intestine. Mushroom polysaccharides can be uptake into Peyer’s patches via M cells and stimulate resident immune cells such as macrophages, DCs, and T-cells (77), thus triggering a cascade of immune responses. Moreover, mushroom polysaccharides can be fermented by gut microbiota in the colon to produce SCFAs, although differently structural mushroom polysaccharides might induce differential production of SCFAs (66). SCFAs play anti-inflammatory activity through inducing the proliferation and differentiation of Foxp3+ regulatory T-cells and the secretion of IL-10 (78). In DSS-induced colitis mice, TFPS could inhibit colonic inflammation via promotion of Foxp3+ T-cells and production of anti-inflammatory cytokines (79). Therefore, mushroom polysaccharides could exert anti-inflammatory actions through multiple pathways but in concert by integrating various receptors-involved signaling within mucosal immune system (Figure 3).

A β-glucan isolated from the fruiting body of *A. blazei* Murill, named ABMP, with a triple-helical structure and a molecular weight of 12.26 kD, showed protective effects on acute inflammatory injury mice established by high dosage zymosan (ZY)-induced organ dysfunction syndrome (MODS) partially through activating NF-κB signaling pathway and then reducing expression of inflammatory cytokines, including TNF-α, IL-1β, IL-6, COX-2, and PGE-2 (48). The effects of a *G. lucidum* spore polysaccharides (BSGLP) with a molecular weight of 26.0 kDa on metabolic disorders and chronic inflammation were evaluated in a mouse model of dietary-induced obesity. BSGLP dramatically reduced serum levels of TNF-α, IL-1β, and monocyte chemoattractant protein-1 (MCP-1), and inhibited macrophage infiltration into white adipose tissues (WAT). Molecular mechanism analysis in mRNA and protein levels showed that BSGLP partially inhibits inflammation via TLR4/Myd88/NF-κB signaling pathway (35). Besides, BSGLP could increase SCFAs production, in particular acetate and butyrate production, which were associated with increased GPR43 expression and reduced inflammation in WAT. Furthermore, fecal microbiota transplantation (FMT) from HFD with BSGLP markedly decreased serum levels of LPS and TNF-α, and slightly decreased the content of IL-1β and MCP-1 in serum in the recipient mice, demonstrating that the anti-inflammatory effects of BSGLP might be in part dependent on the gut microbiota (35). These results suggested that multiple pathways may exist for each mushroom polysaccharides to exert anti-inflammatory effects, however, identifying the major action pathway is still great challenges to researchers. In recent years, a variety of mushroom polysaccharides obtained from different resources have shown anti-inflammation activities in metabolic disorder models (Table 1).

In an acute liver injury mouse model characterized by high levels of alanine aminotransferase (ALT) and aspartate aminotransferase (AST), and high levels of pro-inflammatory cytokines TNF-α, IL-2, IL-6, and MCP-1 induced by lipopolysaccharide/d-galactosamine (LPS/d-GalN), GFP inhibited inflammation level, elevated the levels of SOD and glutathione, and attenuated liver injury. Further analysis revealed that the expression levels of miR-122, a key molecule targeting Nrf2, was reduced and Nrf2/antioxidant response element (ARE) signaling pathway was regulated in liver tissue evidenced by upregulation of transcription factors Nrf2, Nqo-1, and HO-1, and downregulation of transcription factor Kelch-like ECH-associated protein 1 (Keap-1) (80). Keap-1 is vital for maintaining intracellular redox homeostasis and modulating inflammation. HO-1 plays a role in anti-inflammation (81). Thus, Nrf2/ARE signaling pathway might play key roles in modulating the antioxidant response and inflammatory response within cells. This provides an explanation that GFP exhibits anti-inflammation and anti-oxidation effects on the liver injury mouse model partially through activating the miR-122-Nrf2/ARE pathway (80, 81).

However, not all mushroom polysaccharides show consistently beneficial effects on hyperlipidemic animals. For example, *Cordyceps sinensis* polysaccharides (CSP) with a molecular weight of 6.5 × 10^4^ Da could decrease serum levels of LDL-C and TC, but increase serum TG level in HFD-induced obese mice. Surprisingly, CSP deteriorated liver fibrosis and steatosis characterized by increased liver weight and fat accumulation, elevated alanine aminotransferase and infiltration of inflammatory cells in the liver. Furthermore, CSP could increase insulin resistance and inflammation. Therefore, CSP would lead to non-alcoholic steatohepatitis (NASH) and elevate the risk of type 2 diabetes. However, CSP could protect barrier function characterized by an increase in the expression of ZO-1 and occludin in HFD-induced obese mice (82). For these controversial findings, further investigations are needed to clarify the effects and its mechanisms of CSP on metabolic disorders.

### 4.4 Modulating of gut microbiota

**FIGURE 4:**
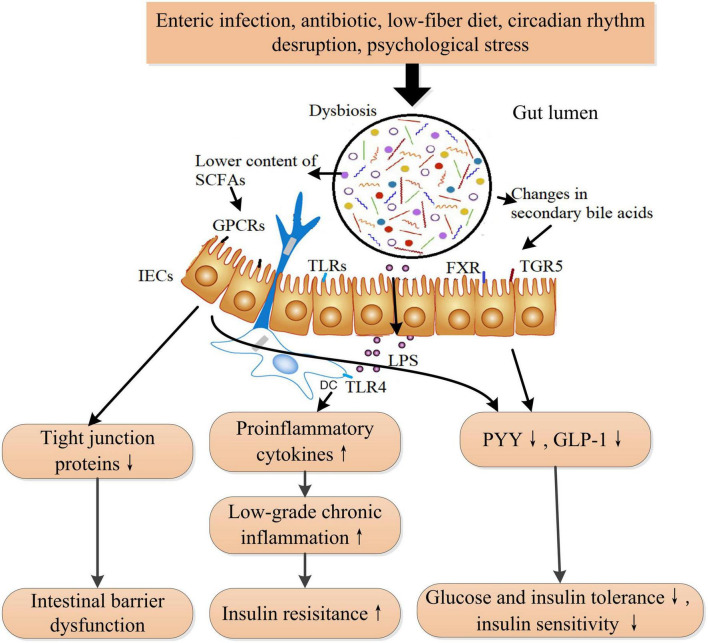
The proposed mechanism of dysbiosis in the gut microbiota that induces insulin resistance and ultimately DM. Gut microbiota is influenced by diet, medication, lifestyle, and stress. Dysbiosis in gut microbiota leads to increased circulating LPS, lower short-chain fatty acids (SCFAs) content, and changes in secondary bile acids. Increased LPS induces low-grade inflammation and insulin resistance through activating the toll-like receptor 4 signaling pathway. Bile acids are transformed into secondary bile acids through the metabolism of the altered gut microbiota. The alterations in secondary bile acids result in reduced GLP-1 and peptide YY secretions through TGR5 and FXR receptors-involved pathways from intestinal L cells. Lower SCFAs also reduces the secretions of GLP-1 and PYY and the expression of tight junction proteins through inactivating GPCRs (GPR41, GPR43, and GPR109A) signaling pathway, leading to intestinal barrier dysfunction and decreased insulin sensitivity. SCFAs also play a role in immune cells. LPS, lipopolysaccharide; TLRs, toll-like receptors; TLR4, toll-like receptor 4; FXR, farnesoid X receptor; TGR5, takeda G protein-coupled receptor 5; PYY, peptide YY; GLP-1, glucagon-like peptide-1; SCFAs, short-chain fatty acids; GPCRs, G-protein coupled receptors. ↑ Indicates increase and ↓ indicates decrease.

Diabetes mellitus is strongly linked and associated with alterations in gut microbiota and intestinal barrier function (83). In patients with type 2 diabetes, Firmicutes and Clostridia are obviously reduced, while Proteobacteria and Bacteroidetes are increased, compared with those in healthy individuals (84). Elevated Ruminococcus and Fusobacterium, whereas decreased Roseburia and Clostridium, as well as fewer butyrate-producing bacteria, were observed in T2DM patients and prediabetes (85, 86). In animal models, continuous intake of a high-fat diet induces dysbiosis in the gut microbiota and intestinal barrier dysfunction, which is linked to the development of DM (32, 87). Gut microbiota involves in modulating insulin sensitivity and glucose tolerance through bile acids metabolism, amino acid metabolism, and SCFAs metabolism. Studies suggested that the absence of the commensal bacterium *Akkermansia muciniphila* damaged to integrity of intestinal barrier and increased intestinal permeability, leading to insulin resistance ultimately in mice and macaques (88). Correspondingly, supplementation of *A. muciniphila* restored normal insulin sensitivity in diabetic mice and macaques (88). Fecal microbiota transplantation from T2DM patients impaired insulin sensitivity and OGT in recipient mice by altering the ability of intestinal microbiota to metabolize bile acids (BAs) and regulating the BAs/glucagon-like peptide-1 (GLP-1) pathway (89). Branched-chain amino acids (BCAA) play important roles in regulating metabolism of glucose, lipid, and protein, except for being utilized as materials for synthesis of proteins (90). Increased BCAA levels contribute to the pathogenesis of insulin resistance and DM in human, whereas enhancing BCAA catabolism improves glucose homeostasis and ameliorates metabolic disorders related complications such as cardiovascular damage (91, 92). Interestingly, an intestinal microbiome possessing a higher potential for biosynthesis of BCAA was associated with increased levels of BCAA in plasma and decreased insulin sensitivity in human (93). Specifically, the gut bacteria *Prevotella copri* could elevate circulating levels of BCAAs and induce insulin resistance in mice (93). Thus, the downstream signaling activated by altered microbial metabolites and microbiota composition promotes the pathogenesis of metabolic diseases such as obesity and diabetes (94, 95) (Figure 4). In addition, LPS derived from the cell wall of Gram-negative bacteria in the gut activates inflammatory response through binding to toll-like receptor 4 (TLR4) on the mucosal immune cells, which is vital for maintaining gut microbiota homeostasis in normal physiology. However, dysbiosis results in elevated level of LPS in gut and therefore initiates low-grade chronic inflammation, which could induce insulin resistance to host (83).

Recently, the modulatory properties of mushroom polysaccharides on gut microbiota have attracted intensive investigations because of the development of metagenome sequencing technology and genome-wide association studies. The genomes of gut microbiota encode a number of carbohydrate-active enzymes (CAZymes), which can degrade mushroom polysaccharides. Three extracellular degradation patterns of polysaccharides by gut microbiota have been illustrated to date, such as starch utilization system (Sus)-like system, ABC-transporter system, and multi-enzyme complex system (66, 96). Mushroom polysaccharides can arrive the colon, where the gut microbiota metabolizes them with mainly SCFAs as the final products through CAZymes degradation systems since gastric juices couldn’t hydrolyze them (97). According to the currently available data, different mushroom polysaccharides exhibit distinct effects on gut microbiota composition and its metabolites (66, 97), resulting in differential physiological responses to the host (98). These may be due to the diverse structures of mushroom polysaccharides (99), the physiological status of the host, and individual differences in gut microbial colonization (100). For example, two kinds of *Flammulina velutipes*-derived polysaccharides with different monosaccharide compositions showed differential productions of SCFAs in rats (101, 102). A heteropolysaccharides derived from *Lentinus edodes* reduced the diversity and evenness of gut microbiota in adult mice while increased the diversity and evenness of gut microbiota in aged mice (103, 104).

Increasing evidences have demonstrated the critical roles of gut microbiota in the prevention and treatment of DM (95). Altering the gut microbiota composition to improve insulin sensitivity and glucose tolerance has received attention from many researchers. Generally, mushroom polysaccharides decrease the ratio of Firmicutes to Bacteroidetes and increase the diversity and richness of gut microbiota along with ameliorations in blood glucose level and insulin sensitivity in obese and diabetic animal models. However, considerable inconsistent impacts on intestinal bacteria at family and genus levels were observed in different studies (Table 1). A polysaccharides from *I. obliquus* decreases FBG, improves intestinal barrier function, and restores the disrupted gut microbiota, characterizing upregulation of Ki-67, ZO-1, and MUC2 expression in the intestine, a restoration of the ratio of Firmicutes to Bacteroidetes, and enriched beneficial bacteria such as Akkermansia and Lactobacillus after the treatment in diabetic mice (32). However, different mushroom polysaccharides might exert differential impacts on the gut microbiota of diabetes, although exhibiting a similar alleviation in metabolic symptoms. For instance, both *Ganoderma atrum* polysaccharide (PGS) and white hyacinth bean polysaccharide (WHBP) ameliorated FBG and insulin resistance, as well as the levels of TC, TG, and LDL-C in T2DM rats. In common, PSG and WHBP alleviated the reduction of Firmicutes and the increase of Bacteroidetes, decreased the genus Prevotella spp, and elevated the genus Lactobacillus in T2DM rats. However, PSG and WHBP also showed their respective characteristic effects on microbial regulation. Specifically, PSG restricted the increase of Streptococcus spp and Clostridium spp, while WHBP could promote the growth of *Roseburia* spp and inhibit *Turicibacter* spp and *Bacteroides* spp in T2DM rats (33). Interestingly, a recent study revealed that commensal bacteria *Bacteroides intestinalis* and *Lactobacillus johnsonii* enriched by treatment of mushroom polysaccharides derived from *Lyophyllum decastes* were causally correlated with decreased plasma TG and LDL-C in HFD-induced obese mice through altering bile acid metabolism of gut microbiota (38), demonstrating a potential therapeutic approach of mushroom polysaccharides in the management of DM. Though many mushroom polysaccharides with structural characterization in somewhat context have been investigated in obese and diabetic models regarding the impacts on gut microbiota, the relationship of the structure of mushroom polysaccharides and the changes of gut microbiota in these models is still poorly understand due to heterogeneity in experimental protocols since environmental factors and animal species strongly affect the initial composition of gut microbiota (105). These influencing factors should be considered in future studies when uncovering the structure-function relationship in terms of mushroom polysaccharides and gut microbiota.

Mushroom polysaccharides show prebiotic properties *in vitro* fermentation, acting as a selective substrate for beneficial gut microbiota growth. The degree of proliferation of Bifidobacterium and Lactobacillus is commonly utilized to assess the prebiotic properties. Seven crude polysaccharides extracted from seven edible mushrooms: *A. auricula-judae*, *L. edodes*, *Pleurotus citrinopileatus*, *Pleurotus djamor*, *P. ostreatus*, *P. ostreatus* (Jacq.Fr.) Kummer and *Pleurotus pulmonarius* have been determined based on probiotic growth promotion (*Lactobacillus acidophilus* and *Lactobacillus plantarum*), pathogenic inhibition (*Bacillus cereus*, *Escherichia coli*, *Salmonella Paratyphi*, and *Staphylococcus aureus*). These results suggested that the monosaccharide composition of crude polysaccharides might influence their prebiotic properties (106). The indigestible residues of five strains of *Macrocybe crassa* mushroom varied in carbohydrate and phenolic content and showed differential impacts on the promotion of the growth of lactic acid bacteria and the inhibition of pathogenic bacteria growth (107). Polysaccharides from mushroom *Clitocybe squamulosa* (CSFP) were fermented in a simulated human fecal microbial model after its treatment by simulated saliva-gastrointestinal digestion. The production of total SCFAs was increased due to an increase in the content of acetic acid, propionic acid, and n-butyric acid, and the ratio of Firmicutes/Bacteroidetes was decreased. Specifically, CSFP promoted the growth of some beneficial bacteria, such as Bacteroides, and Prevotella_9, while inhibited the growth of the typical intestinal pathogen *Escherichia-shigella* (108). This demonstrates that simulated human intestinal fermentation *in vitro* model might be an appropriate tool to investigate the modulatory properties of different mushroom polysaccharides on gut microbiota (109). Variation in the molecular structure such as molecular weight, conformation, and branch degree of mushroom polysaccharides has a significant impact on their solubility, viscosity, and rheological properties (110), which might affect the accessibility of polysaccharides to degrading bacteria in the gut. However, the mechanism of how gut bacteria degrade and utilize mushroom polysaccharides is rarely illustrated, and more researches on this topic are needed.

## 5 Extraction method

**FIGURE 5:**
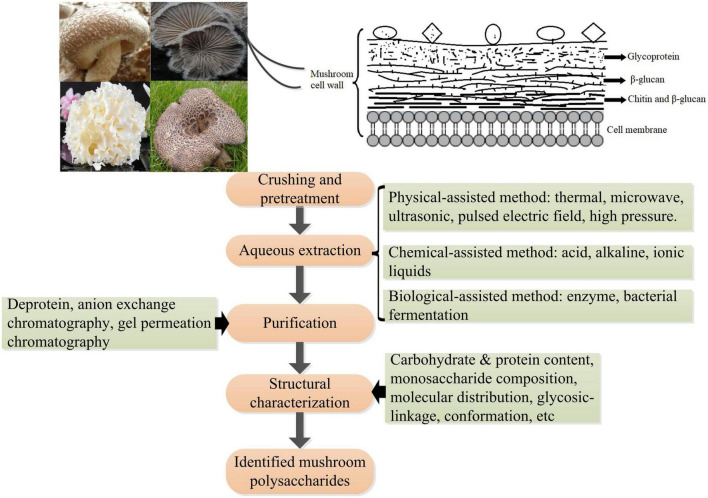
Schematic diagram of the polysaccharides’ location in the cell wall of mushrooms and the procedure of extraction, purification, and structural analysis of mushroom polysaccharides.

The cell wall of mushrooms is a complex consisting mainly of glycoproteins, glucans, chitin, and less proteins; however, the assembly of these molecules has not been fully elucidated. Polysaccharides (50–60%) are the major structural components of the cell wall of mushrooms. Mushroom polysaccharides present in the cell wall can mainly be classified into three layers according to their distribution and cellular localization: (1) the outside layer is glycoprotein consisting of protein and heteropolysaccharides that vary in proportion and monosaccharide composition between species and sites of mushrooms; (2) the middle layer is mainly composed of β-glucan which are insoluble; (3) the inner layer is composed of a complex of chitin and β-glucan (43, 69). With the increasing interest in exploring the structures and bioactivities of mushroom polysaccharides by global researchers, various extraction methods followed by a purification process and structural identification have been developed (111) (Figure 5). Generally, aqueous solution is widely applied to extract polysaccharides with or without pre-treatment with organic solvents (e.g., ethanol, acetone) to remove lipids, and phenols, which facilitates the separation of polysaccharides from other compounds in the cell wall (112). Hot water is the most widely used because of its low cost and ease of handling. The frequently extracted polysaccharides are heteropolysaccharides-proteins complexes present in the outer layer of the cell wall because of its low efficiency in breaking through and penetrating the outer layer of glycoprotein structures. An acid or alkaline solution is commonly used to obtain a high amount of β-glucan present in the middle layer. For example, β-glucans with globular small particle sizes were extracted from the mushrooms *Lentinula edodes* and *P. ostreatus* using an acid-base treatment followed by boiling with 0.5 M NaOH (113). β-D-glucans obtained from mushroom *Macrocybe titans* were consisted of (1→3)-linked β-D-Glc*p* by alkaline extraction method (114). To increase the yield of polysaccharides and improve the efficacy in extraction and/or explore green processes, many novel assisted extraction technologies have been developed. Novel technologies such as microwave-assisted extraction (MAE), ultrasonic-assisted extraction (UAE), enzyme-assisted extraction (EAE), subcritical water extraction (SWE), pulsed electric field-assisted extraction, high pressurized water extraction, deep eutectic solvent extraction, and combined methods such as ultrasonic-microwave synergistic extraction (UMSE), subcritical and pressurized hot water extraction, pressure-associated hot water extraction have emerged in the extraction of mushroom polysaccharides (115–118). These novel extraction technologies mainly help solvent to penetrate through and rupture the outer layer to reach the inner cell wall, contributing to the release of polysaccharides from the compact matrix. However, every extraction method has advantages and disadvantages, such as extraction time, efficiency, and risk of glycosidic bond breakage. Comprehensive information including process parameters, yields, and structural characteristics of obtained polysaccharides on traditional extraction methods, and innovative and advanced extraction methods in the extraction of polysaccharides from mushrooms can refer to relating topic reviews (117, 118). The bioactivities of mushroom polysaccharides greatly depend on their structures, such as molecular weight, monosaccharide composition, branch of degree, and conformation (119), whereas the structures of mushroom polysaccharides vary remarkably based on resources (species), extraction methods, as well as purification processes (120), even dry processes of the fruiting body (121). Thus, novel extraction methods should be evaluated in terms of efficacy, yield, and bioactivity for better assessing the practicability in industrial applications. Recent studies have shown that innovative extraction methods can not only improve the efficacy in the extraction of polysaccharides from natural materials, but also enhance the bioactivities of the polysaccharides obtained (122), which provide a promising direction for future studies.

## 6 Future perspective

**FIGURE 6:**
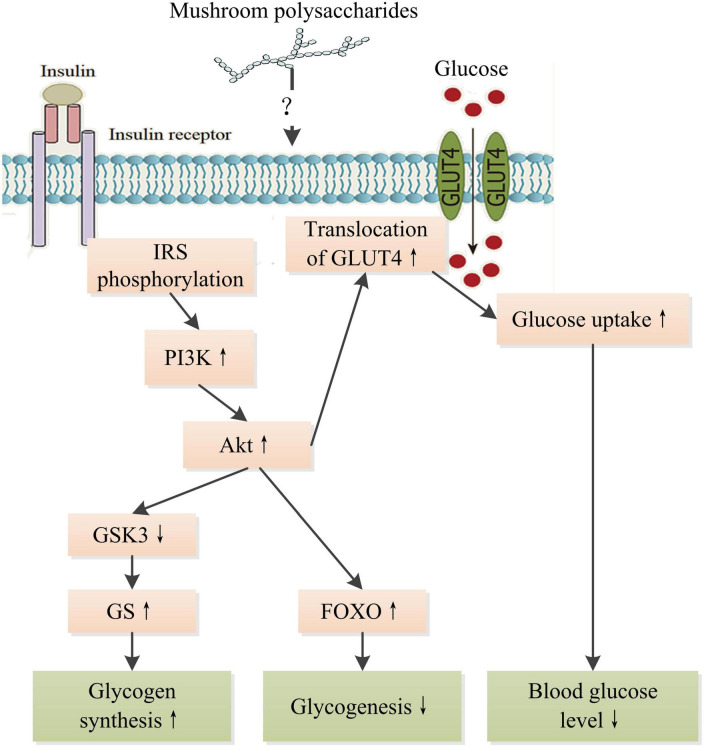
Schematic representation of the hypoglycemic mechanism of mushroom polysaccharides by modulating IR signaling pathway. IR, insulin receptor; IRS, insulin receptor substrate; PI3K, phosphatetidylinostitol-3-kinase; Akt, protein kinase B; GSK3, glycogen synthesis kinase 3; GS, glycogen synthase; GLUT4, glucose transporter 4; FOXO, fork-head box O family proteins. ↑ Indicates increase and ↓ indicates decrease.

In recent decades, many studies have demonstrated that mushroom polysaccharides could modulate the insulin signaling pathway to display hypoglycemic and hypolipidemic effects. Regulation in mRNA and protein levels and phosphorylation of many key molecules such as insulin receptor substrate (IRS), protein kinase B (PKB), phosphatidylinositol-3-kinase (PI3K), protein kinase B (Akt), Jun N-terminal Kinase 1 (JNK), MAPK, and fork-head box O family proteins (FOXO) involved in insulin signaling pathway were observed in diabetic animals treated with polysaccharides extracted from a number of mushrooms such as *Grifola frondosa*, *L. edodes*, *P. linteus*, and *Ophiocordyceps sinensis* (18, 123–125). Lowered blood glucose level, increased glycogen level, and amelioration in damages to tissues were observed as results of treatments as well as improved insulin intolerance. Mushroom polysaccharides might directly interact with insulin receptor or indirectly interplay with insulin signaling through NF-κB pathway and subsequently stimulate cascade signaling to promote glucose uptake of cells. A heteropolysaccharides obtained from *G. frondosa* with an average molecular weight of 66.1 kDa significantly upregulate glucose transporter 4 to improve glucose uptake in insulin resistant HepG2 cell induced by dexamethasone through activating insulin receptor substrate 1 (IRS-1)–PI3K–c-JNK signaling pathway (124). Alkaline soluble polysaccharides derived from the fruiting bodies of *Amillariella mellea*, *Gomphidius rutilus*, and *Agrocybe cylindracea*, respectively, showed better promotion of glucose uptake than those from *Hypsizygus marmoreus*, *Pleurotus eryngii*, and *P. ostreatus* extracted with the same method in Hepa1–6 cells insulin resistance model through activating insulin receptor (IR)-AKT signaling pathway (26). However, there is still a great gap in understanding the detailed interrelation between the intervention of mushroom polysaccharides and the regulation of insulin signaling pathway (Figure 6). Therefore, more works are needed to bridge the gap and reveal the intricate interactions of intracellular signaling pathways within cells, including insulin signaling pathway, MAPK/NF-κB pathway, and oxidative stress-related signaling pathways. The progress in understanding the molecular mechanism of mushroom polysaccharides in modulating insulin signaling pathway would provide a basis for developing targeted therapy for the prevention and management of DM.

The present state of knowledge on the structures of mushroom polysaccharides and their biological functions of anti-diabetes is limited. The successful development and application of mushroom polysaccharides in the anti-diabetic field requires illustrating the structure-activity relationship of mushroom polysaccharides. Considering the great variability in monosaccharide composition, molecular weight (molecule size), glycosidic linkage, and conformation of mushroom polysaccharides within individual species, not all mushroom polysaccharides contained in mushrooms exhibit equivalently therapeutic activity. For example, six alkaline soluble polysaccharides extracted from the fruiting bodies of *A. mellea*, *G. rutilus*, *A. cylindracea*, *H. marmoreus*, *P. eryngii*, and *P. ostreatus*, respectively, showed differential improvement of insulin sensitivity in the Hepa1–6 insulin resistance cell model, suggesting that the specific structure of polysaccharides could play an important role in modulating insulin sensitization (26). However, there is insufficient data to conclude which structural characteristics are key determinants of mushroom polysaccharides in anti-diabetes, and which type of mushroom polysaccharides might have better effects. With the expanding exploration of mushrooms, more and more mushroom species are found and identified. Given the great potential of mushroom diversity, and structural complicity and diversity of mushroom polysaccharides, the current lacking of internationally recognized standard protocols for the testing anti-diabetic activity would bring considerable challenges to their translational applications for scientists worldwide. Future studies assessing the efficacy of mushroom polysaccharides with precise structural characterization in well-recognized models for the prevention and/or treatment of DM are needed. Furthermore, deep uncovering the anti-diabetic mechanisms using modern technologies, including metagenomics, proteomics, metabolomics, and transcriptomics, would expand our understanding of the biological and pharmacological properties of mushroom polysaccharides. These would lead to the successful development of therapeutic agents in managing DM.

There has been an increasing interest in developing synergic foods as functional foods or nutraceuticals, which have advantages in stronger health benefits and convenience for the management of non-transmissible chronic diseases (126). Besides mushroom polysaccharides, other active substances such as minerals and trace elements also have beneficial roles in insulin resistance and DM (127). Thus, mushroom polysaccharides combined with other active components aiming at synergistic effects would provide a potentially powerful approach to preventing and treating DM and its complications. Mushroom polysaccharides show a good metal ion chelating ability (43). Some metal ions are vital for metabolism. For example, chromium (III) is an essential trace element and plays a vital role in maintaining insulin sensitivity and glucose homeostasis in the human body (128). Polysaccharides-metal ion complexes to improve the therapeutic activities in anti-diabetes have been conducted. For instance, chromium (Cr)-*G. frondosa*-derived polysaccharides complexes have been synthesized and evaluated in terms of their anti-hyperlipidemic and anti-hyperglycemic effects in HFD- and STZ-induced diabetic mice. The Cr-polysaccharides complex showed better activities in reducing blood glucose level, improving liver glycogen synthesis, and decreasing serum TC and TG than polysaccharides alone, suggesting outstanding therapeutic actions of the complex in improving glucose and lipid metabolism in diabetes (129). Selenium (Se) is a vital micronutrient and plays a crucial role in redox homeostasis in the body (130). Se-rich polysaccharides extracted from the fruiting body of *C. militaris* exhibited stronger anti-inflammation and anti-lipidemic properties than the same source of Se-deficient polysaccharides (29). Furthermore, a combination of inulin and *G. lucidum* polysaccharides displayed better activities in improving insulin sensitivity, increasing glycogen synthesis, and ameliorating lipid metabolism than inulin or *G. lucidum* polysaccharides alone in T2DM rats, demonstrating synergistic actions of inulin and *G. lucidum* in anti-diabetes (131).

In addition, active polysaccharides combined with nano-and micro-technological strategies or functionalized with specific molecules would improve their efficacy in anti-diabetes through targeting delivery and/or increasing intestinal permeability (132). A polysaccharide-based micelle-hydrogel containing insulin and nattokinase was developed. This synergistic therapy system not only possesses glucose-responsive insulin delivery properties, but also provides good thrombolytic capacity (133). Se-*C. ventricosum*-derived polysaccharides nanoparticles exhibited significantly higher anti-diabetic effects than Se nanoparticles alone (134). Therefore, a synergistic formula composed of multiple mushroom polysaccharides and/or other active components with different pharmacological properties by targeting multiple action pathways combined with efficient delivery technology would synergistically improve the outcomes in the prevention and management of DM and its complications.

Increasing studies focus on the interventions of mushroom polysaccharides in diabetic animal model. However, little attention has been paid to the pharmacological actions of mushroom polysaccharides in DM patients. Available data on human mainly use mushroom powder or mushroom extracts. In a single-blind trial, 24 T2DM patients were randomized for consumption of 50 mg/kg BW of dried and powdered *P. ostreatus* or *Pleurotus cystidiosus*, respectively (135). Both mushroom interventions effectively reduced postprandial serum glucose levels and increased postprandial serum insulin levels of diabetes patients. Twenty-two subjects with impaired glucose tolerance consumed a meal either fortified with 20 g of dried *P. ostreatus* powder (contained 8.1 g β-glucan) or without enrichment, a fortified meal improved the levels of postprandial GLP-1 and non-esterified free fatty acids compared to control meal in a double-blind, randomized controlled crossover trial, suggesting the beneficial effects of mushrooms β-glucan in improving postprandial metabolism for peoples with a high risk of development of T2DM (136). A possible reason might be that it is tough and time-consuming to prepare a sufficient amount of purified mushroom polysaccharide used in clinical trials. This obstacle requires cooperation between institutes and industrial companies. Further studies and well-controlled clinical trials should be conducted in this context to develop the most reliable anti-diabetic agents screened from mushroom polysaccharides.

## Author contributions

XL and DL: conceptualization, data curation, investigation, visualization, and writing—original draft, review, and editing. JG and JC: writing—original draft, review, and editing. XX: conceptualization, supervision, methodology, formal analysis, project administration, funding acquisition, resources, and writing review and editing. All authors contributed to the article and approved the submitted version.
